# Neuroprotective Effects of Ultra-Low-Molecular-Weight Heparin on Cerebral Ischemia/Reperfusion Injury in Rats: Involvement of Apoptosis, Inflammatory Reaction and Energy Metabolism

**DOI:** 10.3390/ijms14011932

**Published:** 2013-01-17

**Authors:** Zhi-Guo Zhang, Xin Sun, Qing-Zhu Zhang, Hua Yang

**Affiliations:** 1 Department of Pharmacy, the 88th Hospital of PLA, Hushan East Road, Tai’an 271000, Shandong, China; E-Mails: tazzg@163.com (Z.-G.Z.); zzgta@yahoo.com.cn (X.S.); 2 Pharmacological Institute of New Drugs, School of Pharmacy, Shandong University, 44 Wenhua Xi Road, Ji’nan 250012, Shandong, China; 3 Department of Immunology, Taishan Medical College, 2 Yingsheng East Road, Tai’an 271016, Shandong, China; E-Mail: yhuabb@163.com

**Keywords:** ultra-low-molecular-weight heparin (ULMWH), energy metabolism, apoptosis, inflammatory reaction, middle cerebral artery occlusion (MCAO)

## Abstract

Previous experiments showed that ultra-low-molecular-weight heparin (ULMWH) reduced the infarct and neurologic deficit in rats followed by transient cerebral ischemia, but the mechanisms of its neuroprotective effect are unclear. This study reported the effect of ULMWH on energy metabolism, inflammatory reaction and neuronal apoptosis. Male Wistar rats were subjected to middle cerebral artery occlusion (MCAO) for 2 h followed by reperfusion for 24 h. ULMWH (0.5, 1 mg/kg, i.v.) was administered after the MCAO and reperfusion. 24 h after the reperfusion, Spectrophotometric assay was used to determine the activity of ATPase and the content of lactic acid in the brain. The ICAM-1 and Caspase-3 genes were investigated by RT-PCR. Furthermore, the apoptotic percentage of cells in hippocampus was quantified by flow cytometry. Compared with the model group, ULMWH significantly decreased lactic acid content and increased ATPase activity in ischemic brain. At the same time, ULMWH inhibited the neural apoptosis and decreased the expressions of ICAM-1 and Caspase-3 mRNA in hippocampus. These findings suggest that ULMWH exhibits a neuroprotective effect against cerebral ischemia/reperfusion injury, partly through improving energy metabolism, inhibiting apoptosis and attenuating inflammatory reaction.

## 1. Introduction

Ischemic stroke is a devastating disease with a complex pathophysiology. The precise sequence of events leading to neuronal death following stroke is at present not fully defined. However, a core picture is developing. Brain is almost exclusively dependent on the continuous steady flow of glucose and oxygen to undergo oxidative phosphorylation for energy production, because it has no stores of energy. The disruption of blood flow causes accumulation of lactic acid via anaerobic glycolysis, and the cellular ATP decreases. The reduction of cellular energy results in the impairment of vital cellular functions, such as the activity of ATPase, which contribute to Ca^2+^ overload. Then, the increase of intracellular Ca^2+^ concentration leads to cell damage and eventual death. Energy depletion and necrotic neuronal death can trigger the inflammatory injury. Evidence suggests that necrosis and apoptosis are the main characteristics of neuronal death following acute cerebral ischemia-reperfusion injury [1].

Several studies have shown the neuroprotective effects of ULMWH. ULMWH can obviously reduce Aβ25-35-induced neurotoxic effects and might act as a potential agent for Alzheimer’s disease [2]. ULMWH can also antagonize glutamate-induced apoptosis in cortical cells through a decrease of Ca^2+^ release and modulation of apoptotic processes [3]. Our previous study [4] showed the beneficial effects of ULMWH on ischemia-reperfusion injury in rats by decreasing infarct, reducing the neurologic deficit, inhibiting Ca^2+^ accumulation, decreasing MDA content and increasing SOD activity in ischemic brain. Administration of ULMWH significantly decreased neurological deficit scores and showed a much better potency compared with the treatment with LMWH, both at the dose of 1 mg/kg (ULMWH, 0.7 ± 0.67; LMWH, 1.6 ± 0.52; model, 1.8 ± 0.42). In this study, using the transient ischemia model, we tested whether ULMWH can improve the energy metabolism, affect the inflammatory reaction and inhibit the apoptosis in rats subjected to the occlusion of the middle cerebral artery.

## 2. Results and Discussion

### 2.1. Effects of ULMWH on ATPase Activity and LD Content in Brain of Ischemic Cortex

As shown in Table 1, ATPase activity was much lower and lactic acid level was higher in the model control group than those in the sham control group. The treatment with 0.5, 1 mg/kg of ULMWH or 1 mg/kg of LMWH increased ATPase activity in brain tissue of the focal cerebral ischemic rats compared with the model control group (*p* < 0.05 or 0.01). At the same time, the level of lactic acid was significantly reduced (*p* < 0.05 or 0.01).

The ischemic brain injury is caused by a complex interplay between different factors and signal cascades in the nervous cells leading to degeneration. Acute cerebral ischemia firstly results in energy metabolism failure with a sharp decrease in formation of adenosine triphosphate (ATP) [5] and excessive accumulation of lactic acid [6,7]. ATP is a critical energy source for maintaining the ion pumping of Na^+^–K^+^–ATPase and Ca^2+^–Mg^2+^–ATPase, which regulates the ionic concentration gradients necessary to generate action potentials by neurons. The early stage of energy metabolism failure in cerebral ischemia injury can be studied by measuring the level of lactic acid and the activity of ATPases. In this study, we found that energy metabolism failure occurred in the cortex because of the shortage of oxygen and glucose. As a result, lactic acid accumulated, and the activity of Ca^2+^–Mg^2+^–ATPase decreased in the ischemic brain tissue. Treatment of ULMWH reversed these changes, indicating its protective effects on improving energy metabolism.

### 2.2. Quantification of Apoptosis by Flow Cytometry

Compared with the sham control group, the apoptotic rate of the model control group had significant changes, increasing to 20.3% ± 7.95% (*n* = 9, *p* < 0.01, Table 1). The treatment with 0.5, 1 mg/kg of ULMWH or 1 mg/kg of LMWH decreased the percentage of apoptosis compared with the model control group (*p* < 0.05 or 0.01). ULMWH at the dose of 1mg/kg decreased the rate to 6.9% ± 2.32% and showed a better effect compared with LMWH at the same dose (*p* < 0.05).

During cerebral ischemia, a very severe ischemic circumstance causes rapid and irreversible destruction of cells, whereas mild ischemic insult undergoes apoptosis [8]. The majority of cells in the ischemic brain that undergo apoptosis are neurons [9,10]. Apoptosis primarily occurs in the penumbra at 6 h and then sharply increases in the ischemic rim and core areas at 24–48 h post-ischemia [11,12]. In the present study, the percentage of apoptosis was increased after 24 h of reperfusion in ischemic hippocampus. Treatment with ULMWH significantly decreased the rate.

### 2.3. Semi-Quantitative RT-PCR of ICAM-1 and Caspase-3

**Figure 1:**
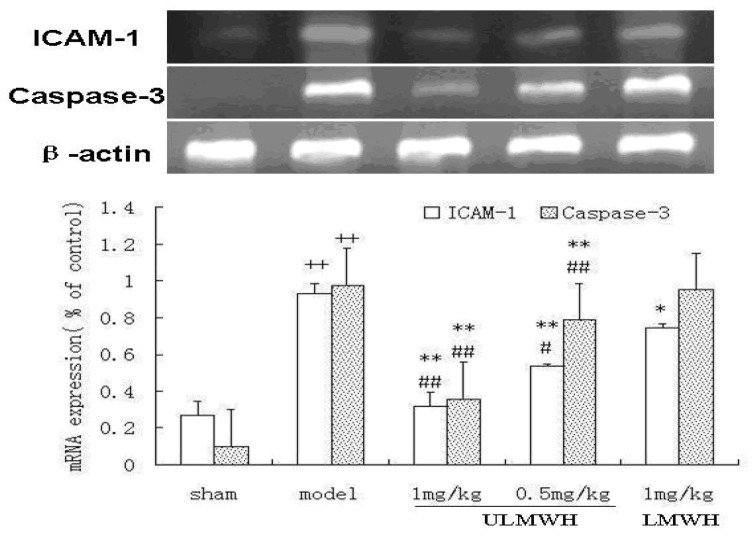
The expression of ICAM-1 and Caspase-3 mRNA in the ischemic hippocampus. Values are expressed as mean ± SD. (*n* = 9). Significance was determined by ANOVA, followed by Dunnett’s test. ^++^*p* < 0.01 compared to the sham control group; * *p* < 0.05, ** *p* < 0.01 compared to the model control group; ^#^*p* < 0.05, ^##^*p* < 0.01 compared to the LMWH-treated group.

The results showed that the expression levels of ICAM-1 and Caspase-3 mRNA were higher in the model control group than in the sham control group (*p* < 0.01, Figure 1). The treatments with ULMWH decreased their expressions significantly. ULMWH had a better effect compared with LMWH at the same dose.

Inflammation is increasingly recognized to be the key element in pathological progression of ischemic stroke. While effective reperfusion of the occluded cerebral vessel is the goal of thrombolytic therapy, both spontaneous and therapeutic reperfusion are associated with inflammatory responses based on the cerebral vasculature, which might paradoxically contribute to cerebral injury [13]. It has been shown that the expression of ICAM-1 mRNA is remarkably increased in the ischemic area at 1–4 h of reperfusion in transient MCAO models [14]. It plays a pivotal role in the infiltration of leukocytes into the brain parenchyma after stroke and may represent important therapeutic targets. Several studies have now shown that blocking ICAM-1 with antibodies [15–17] or inhibiting ICAM-1 mRNA with antisense oligonucleotide [18] improves the outcome from experimental stroke. Similarly, mice deficient in ICAM-1 had smaller infarcts compared to wild-type mice [19,20]. In the present study, there was a significant up-regulation of ICAM-1 mRNA in ischemic hippocampus, while ischemia-induced ICAM-1 mRNA expression was significantly reduced by ULMWH 24 h after reperfusion.

Recent researches verified that the central component of the machinery of apoptosis is a proteolytic system involving a family of proteases called cysteine aspartase (caspase). Caspase played an important role in apoptosis and might be a final executant of apoptosis. Different kinds of caspase participated in a cascade that is triggered in response to proapoptotic signals and culminates in cleavage of a set of proteins, resulting in disassembly of the cell. Caspase-3, a pivotal factor of apoptosis, was activated via a series of caspase cascade reactions and then activated deoxyribonuclease, which cleaves DNA to fragments. In the present study, there was a significant upregulation of caspase-3 mRNA in ischemic hippocampus, while ischemia-induced caspase-3 mRNA expression was reduced by ULMWH 24 h after reperfusion.

## 3. Experimental Section

### 3.1. Chemicals

ULMWH (mean MW = 2200) and LMWH (mean MW = 5500) were all sodium salts provided by the Institute of Biochemical and Biotechnological Drugs, School of Pharmacy, Shandong University. Lactic acid and ATPase assay kits were purchased from Nanjing Jiancheng Bioengineering Institute (Nanjing, China).

### 3.2. MCAO-Induced Transient Focal Cerebral Ischemia in Rats

Male Wistar rats (weighing 250–280 g) were allowed food and water *ad libitum*, and the room temperature was kept at 26~28 °C. All of the animal experiments were carried out in accordance with the National Institutes of Health Guide for the Care and Use of Laboratory Animals, and the protocols were approved by the Committee for Animal Research at Shandong University. Before undergoing the experimental procedures, the animals were clinically normal and free of apparent infection or inflammation and showed no neurological deficits. Animals were subjected to MCAO, as described previously [4].

### 3.3. Drug Treatment

The animals were randomly divided into five groups each containing 18 animals: model control; ULMWH-treated group (1 and 0.5 mg/kg, i.v.); and LMWH-treated group (1 mg/kg, i.v.). Besides, 18 rats were used for sham control, which were given the same protocol without inserting the nylon monofilament. Drug was dissolved in saline and intravenously administrated twice. The first injection was administered 10 min after the MCA occlusion; 2 h later (10 min after reperfusion), the next injection was administered.

### 3.4. The ATPase Activity and Lactic Acid Level in Brain Tissue after Injury

Twenty-four hours after reperfusion, 9 rats from each group were anesthetized with sodium pentobarbital (100 mg/kg). The brains were removed from the skull immediately to determine lactic acid level and ATPase activity, the ischemic parietal cortex was homogenized in saline. The 10% homogenate was centrifuged at 3000 rpm and 4 °C for 15 min, and the supernatant was used to determine lactic acid level at 530 nm and ATPase activity at 660 nm, as the methods provided by the assay kits.

### 3.5. Quantification of Apoptosis by Flow Cytometry

A flow cytometric method was used to assess the percentage of apoptotic nuclei [21]. Briefly, the ischemic hippocampus CA1 was isolated and cut into about 1 mm^3^ fragments, which were digested with 0.125% trypsin at 37 °C for 5 min. Trypsinization was discontinued by adding DMEM medium containing 10% bovine serum. The isolated brain cells were filtered through nylon mesh (200 mesh) and washed twice at 1000 rpm for 5 min. The cells were fixed in 70% (*v*/*v*) ethanol overnight at 4 °C. After rewashing with cold PBS, the cells were resuspended in 500 μL of PBS containing propidium iodide (PI) (40 μg/mL) and RNase (100 μg/mL). PI staining was used to determine the percentage of cells in different phases of the cell cycle. Cell cycle analysis was performed by using a Becton-Dickinson fluorescence-activated cell analyzer and data analysis was performed with Modifit LT 2.0 (Becton-Dickinson, San Jose, CA, USA). Sub-G_1_ peaks were used to calculate the apoptotic rate of the cells.

### 3.6. Semi-Quantitative RT-PCR of ICAM-1 and Caspase-3

Total RNA for RT-PCR analysis was extracted from the ischemic hippocampus CA1 using Trizol reagent, according to the manufacturer’s instructions (Invitrogen, Carlsbad, CA, USA). The primers for intercellular adhesion molecule-1 (ICAM-1) RT-PCR were: forward, 5′-AGA CAC AAG CAA GAG AAG AA-3′; and reverse, 5′-GAG AAG CCC AAA CCC GTA TG-3′ [22]. The primers for Caspase-3 RT-PCR were: forward, 5′-CAG CTC GCA ATG GTA CCG AT-3′; and reverse, 5′-GCA TTG ACA CAA TAC ACG GG-3′ [23]. PCR (polymerase chain reaction) conditions were 30 cycles of denaturation at 94 °C for 45 s, annealing at 55 °C for 45 s and extension at 72 °C for 45 s. PCR products were separated by electrophoresis through 1.5% agarose gel containing 0.l g/mL ethidium bromide and imaged using a image analysis system (JM VISTEC, Suzhou, China). The relative transcript abundance is expressed as the percentage of the target transcript level to that of β-actin.

### 3.7. Statistical Analysis

Data were expressed as mean ± SD. Statistical significance was determined by one-way analysis of variance, followed by Dunnett’s test. All of the statistical analyses were performed by software SPSS for Windows; *p* < 0.05 was considered statistically significant.

## 4. Conclusions

Our data showed that treatment with ULMWH decreased lactic acid level and increased ATPase activity, decreased the expression of ICAM-1, caspase-3 mRNA and antagonized the ischemia/reperfusion-induced apoptosis. ULMWH at a lower dose seems to show a comparable effect as LMWH does. That might help reduce the probability of hemorrhage, which is the main adverse effect of LMWH.

## Figures and Tables

**Table 1:** Effect of ultra-low-molecular-weight heparin (ULMWH) on energy metabolism and neural apoptosis in brain of rats after reperfusion for 24 h.

Group	Dose (mg/kg)	Lactic acid (mmol/g)	ATPase (μmolPi/mg·h)	Percentage of cell apoptosis (%)
Sham	Saline	0.75 ± 0.19 ^**^	3.97 ± 0.60 ^**^	5.6 ± 2.10 ^**^
Model	Saline	1.06 ± 0.18	2.42 ± 0.67	20.3 ± 7.95
ULMWH	0.5	0.89 ± 0.13 ^*^	3.33 ± 0.57 ^*^	11.4 ± 3.70 ^*^
ULMWH	1	0.83 ± 0.09 ^**^	3.49 ± 0.35 ^**^	6.9 ± 2.32 ^**^
LMWH	1	0.85 ± 0.13 ^*^	3.56 ± 0.57 ^**^	10.6 ± 1.77 ^*^

Data are expressed as mean ± SD. (*n* = 9). Significance was determined by ANOVA, followed by Dunnett’s test. Asterisks represent a significant difference between model control group and other groups (^*^p < 0.05, ^**^p < 0.01).
